# RNA-Seq-Based Analysis of Cortisol-Induced Differential Gene Expression Associated with *Piscirickettsia salmonis* Infection in Rainbow Trout (*Oncorhynchus mykiss*) Myotubes

**DOI:** 10.3390/ani11082399

**Published:** 2021-08-13

**Authors:** Rodrigo Zuloaga, Phillip Dettleff, Macarena Bastias-Molina, Claudio Meneses, Claudia Altamirano, Juan Antonio Valdés, Alfredo Molina

**Affiliations:** 1 Laboratorio de Biotecnología Molecular, Facultad de Ciencias de la Vida, Universidad Andres Bello, Santiago 8370186, Chile; r.zuloaga@uandresbello.edu (R.Z.); satryl@veterinaria.uchile.cl (P.D.); jvaldes@unab.cl (J.A.V.); 2 Interdisciplinary Center for Aquaculture Research (INCAR), Concepción 4030000, Chile; 3 Centro de Biotecnología Vegetal, Facultad de Ciencias de la Vida, Universidad Andres Bello, Santiago 8370186, Chile; macarena.bastias@unab.cl (M.B.-M.); claudio.meneses@unab.cl (C.M.); 4 Laboratorio de Cultivos Celulares, Escuela de Ingeniería Bioquímica, Pontificia Universidad Católica de Valparaíso, Valparaíso 2362803, Chile; claudia.altamirano@ucv.cl; 5 Centro de Investigación Marina Quintay (CIMARQ), Facultad de Ciencias de la Vida, Universidad Andres Bello, Valparaíso 2340000, Chile

**Keywords:** stress, *Piscirickettsia salmonis*, salmonid, skeletal muscle cells, RNA-seq

## Abstract

**Simple Summary:**

Skeletal muscle is the most abundant tissue in fish and the main product of the Chilean salmonid aquaculture industry. Intensive farming conditions generate stress and increased susceptibility to infectious diseases, such as salmonid rickettsial septicemia (SRS), that directly affect this tissue. However, the immunocompetence of skeletal muscle during infection is poorly understood. To further explore the interplay between pathogen infection and stress on this tissue, we analyze the transcriptional profile of isolated rainbow trout (*Oncorhynchus mykiss*) muscle cells pretreated with 3 h of the stress hormone cortisol, and then infected with the SRS etiologic agent *Piscirickettsia salmonis* for 8 h, using RNA sequencing technology. For the first time, the obtained data reveals the biological processes related to programmed cell death, negative regulation of cell proliferation, and innate immune response. These results are validated by real-time qPCR. Furthermore, cortisol pretreatment significantly stimulated bacterial gene expression compared to infected cells. These data demonstrated that fish skeletal muscle can activate an intrinsic immune-like response against *P. salmonis* that is differentially regulated by cortisol. The information provided here will help us to understand the molecular mechanisms of fish muscle cells respond to infection, which could prevent *P. salmonis* outbreaks in skeletal muscle under stress conditions.

**Abstract:**

Salmonid rickettsial septicemia (SRS) is the major infectious disease of the Chilean salmonid aquaculture industry caused by *Piscirickettsia salmonis*. Intensive farming conditions generate stress and increased susceptibility to diseases, being skeletal muscle mainly affected. However, the interplay between pathogen infection and stress in muscle is poorly understood. In this study, we perform an RNA-seq analysis on rainbow trout myotubes that are pretreated for 3 h with cortisol (100 ng/mL) and then infected with *P. salmonis* strain LF-89 for 8 h (MOI 50). Twelve libraries are constructed from RNA samples (*n* = 3 per group) and sequenced on Illumina HiSeq 4000. A total of 704,979,454 high-quality reads are obtained, with 70.25% mapped against the reference genome. In silico DETs include 175 total genes—124 are upregulated and 51 are downregulated. GO enrichment analysis reveals highly impacted biological processes related to apoptosis, negative regulation of cell proliferation, and innate immune response. These results are validated by RT-qPCR of nine candidate transcripts. Furthermore, cortisol pretreatment significantly stimulated bacterial gene expression of *ahpC* and *23s* compared to infection. In conclusion, for the first time, we describe a transcriptomic response of trout myotubes infected with *P. salmonis* by inducing apoptosis, downregulating cell proliferation, and intrinsic immune-like response that is differentially regulated by cortisol.

## 1. Introduction

Piscirickettsiosis, also called salmonid rickettsial septicemia (SRS), is one of the most important infectious diseases affecting the Chilean salmonid aquaculture industry [1]. SRS is caused by *Piscirickettsia salmonis*, which is a gram-negative bacterium that is aerobic, non-encapsulated, generally non-motile, pleomorphic, but predominately coccoid, and facultative intracellular [2]. Previous studies have revealed that *P. salmonis* enters immune cells (macrophages) of rainbow trout (*Oncorhynchus mykiss*) and regulates apoptotic processes [3,4]. Among the mechanisms that *P. salmonis* uses to infect are secretion system type 4 deficient in organelle trafficking/intracellular multiplication (Dot/Icm), adhesion molecules (type 4 pili), and the inhibition of reactive oxygen species [5]. This septicemic disease affects a variety of salmonid species, such as Atlantic salmon (*Salmo salar*), Coho salmon (*Oncorhynchus kisutch*), and rainbow trout [6]. Globally, rainbow trout is a salmonid of high commercial interest for the industry, with its skeletal muscle being the most important tissue in terms of aquaculture products [7].

Currently, the aquaculture industry mainly uses intensive farming conditions that generate stress [8]. Stress initially produces and releases hydrocortisone hormone, better known as “cortisol”, the main neuroendocrine regulator of the stress response [9]. Cortisol triggers metabolic and physiological changes in several processes of fishes, particularly in the immune system [10]. The mechanism of cortisol action is related to its interaction with corticosteroid receptors and regulation of stress-sensitive genes [11]. In fish muscle, this signaling pathway promotes an imbalance between protein synthesis and degradation, leading to skeletal muscle atrophy [12].

Previously, in vivo studies have found evidence of the immune response of skeletal muscle during an infectious process [13,14]. Although muscle was recently suggested to be an immunocompetent organ by itself in fish [15], these data could be masked by infiltration of immune cells in skeletal muscle. To demonstrate this, few studies have performed stimulations with lipopolysaccharide [16] or proinflammatory cytokines [17] in salmonid muscle cells. A recent study demonstrated the in vitro ability of rainbow trout myotubes to implement an inflammatory and antimicrobial response against *P. salmonis* [18]. In this sense, isolated and differentiated muscle cells, or myotubes, could be better considered to regulate fish muscle physiology against the systemic effects of infection through in vivo studies [19]. This inflammatory response of skeletal muscle to *P. salmonis* has been further investigated by RNA-seq in an in vivo challenge, revealing interesting details [20]. Moreover, high-throughput mRNA sequencing (RNA-seq) has emerged as an optimal strategy for analyzing global gene expression [21].

In the present work, we performed an RNA-seq analysis in vitro to evaluate whether cortisol-mediated stress produces an effect in rainbow trout myotubes infected with *P. salmonis*. The data demonstrate that cortisol and/or infection induce transcriptomic changes in fish muscle cells and potentially mediate apoptosis, cell proliferation, and the innate immune response. Finally, cortisol pretreatment differentially stimulated bacterial gene expression in infected myotubes.

## 2. Materials and Methods

### 2.1. Primary Culture of Rainbow Trout Myotubes

Healthy *P. salmonis* free-juvenile rainbow trout (weight 9.4 ± 1.8 g; length 10 ± 2 cm) were obtained from Río Blanco “Federico Albert Taupp” Pisciculture (Los Andes, Valparaíso, Chile). Fishes were reared and used according to a protocol approved by the bioethical committee for animal experiments of Universidad Andrés Bello. Muscle cells were obtained from four rainbow trout (5–8 g). The dorsal white skeletal muscle that was disintegrated in Dulbecco’s modified Eagle’s medium (DMEM, #D7777, Sigma-Aldrich, San Luis, MO, USA) containing 9 mM NaHCO_3_, 20 mM HEPES, and 10% donor horse serum (#04-004-1A, Biological Industries, Beit HaEmek, Israel) at pH 7.4, with 100 U/mL penicillin and 100 μg/mL streptomycin (#30-004-CI, Corning Inc., Corning, NY, USA). After mechanical dissociation, the muscle was digested with 0.2% collagenase type II (#17101-015, Thermo Fisher, Waltham, MA, USA) in DMEM for 1 h at 18 °C. The suspension was centrifuged at 300× *g* for 5 min and then digested with 0.1% trypsin-EDTA (#25-053-CI, Corning Inc.) in DMEM for 30 min at 18 °C. After trypsin was deactivated, the suspension was filtered, centrifuged at 500× *g* for 10 min, and finally resuspended in 5 mL of DMEM. The cell suspension was collected to perform cell counts and to validate viability with Trypan Blue 0.2% solution (#T8164, Sigma-Aldrich).

Muscle cells were seeded in 12-well plates (cell density of 8 × 10^5^ per well) previously treated with poly-l-lysine (2 µg/cm^2^, #P5899, Sigma-Aldrich) and laminin (20 µg/mL, #L2020, Sigma-Aldrich). First, cells were incubated at 18 °C for 7 days in DMEM, 9 mM NaHCO_3_, 20 mM HEPES, and 10% fetal bovine serum (#2442, Sigma-Aldrich) at pH 7.4, 100 U/mL penicillin, and 100 μg/mL streptomycin and then differentiated into myotubes by an additional seven days of culture in DMEM, 9 mM NaHCO_3_, 20 mM HEPES, 2% fetal bovine serum, with 100 U/mL penicillin and 10 mg/mL streptomycin. The procedure to obtain rainbow trout myotubes was repeated three times independently (*n* = 3).

### 2.2. Piscirickettsia salmonis Culture

The bacteria *P. salmonis* strain LF-89 (ATCC VR-1361) were grown as previously reported [22]. Briefly, *P. salmonis* was cultivated in basal media composed of yeast extract 2.0 g/L, peptone from meat digested 2.0 g/L, (NH_4_)_2_SO_4_ 1.32 g/L, MgSO_4_·7H_2_O 0.1 g/L, K_2_HPO_4_ 6.3 g/L, NaCl 9.0 g/L, CaCl_2_·2H2O 0.08 g/L, and FeSO_4_·7H_2_O 0.02 g/L. Then, bacteria were grown with orbital shaking (100 rpm) at 23 °C and monitored using turbidimetry at an optical density (OD) of 600 nm.

### 2.3. Experimental Design

The concentration and time used for all treatments were previously standardized by [18]. Briefly, myotubes from each plate were divided into the following four experimental groups: (i) Control (CTRL, medium + cortisol vehicle), (ii) cortisol treatment (CORT), (iii) *P. salmonis* infection (INF), and (iv) cells pretreated with cortisol and then infected with bacteria (C+INF). Before treatments, myotubes were washed once with 1X PBS (phosphate-buffered saline) at pH 7.4. First, myotubes were preincubated with cortisol (100 ng/mL, #H0888, Sigma-Aldrich) or cortisol vehicle (DMSO) in DMEM, 9 mM NaHCO3, and 20 mM HEPES for 3 h at 18 °C. In parallel, *P. salmonis* was quantified by measuring the OD 600 nm to ensure it was between 0.3 and 0.6 (logarithmic phase) using the basal medium, and the McFarland formula was applied [23,24]. Then, bacteria were centrifuged at 5000× *g* for 30 min, the supernatant was discarded, and the cells were resuspended in cell medium. After 3 h of incubation with cortisol or vehicle, the medium was changed. The myotubes were infected with *P. salmonis* at a multiplicity of infection (MOI) of 50 or medium without bacteria for 8 h at 18 °C.

### 2.4. Immunostaining

Immunostaining for *P. salmonis* and rainbow trout myotubes was performed to detect the subcellular location in fish muscle cells. For this, bacteria were detected with a FITC-conjugated antibody (in green), and muscle cells were visualized with an actin cytoskeleton marker (in red) and nuclear staining (in blue). Briefly, myotubes were cultured with a 12 mm coverslip (8 × 10^5^ cells/well) and treated as previously mentioned. Then, the cells were washed with 1X PBS and fixed with 4% paraformaldehyde (diluted with 1X PBS) for 10 min at room temperature (RT). After three washes, cells were permeabilized with 0.1% BSA (bovine serum albumin)–3% Triton X-100 (diluted with 1X PBS) for 30 min at RT. Fixed and permeabilized cells were incubated with anti-*P. salmonis* primary antibody (#D-FP-SRS-IF, Ango, Santiago, Chile) diluted 1:100 in 3% BSA-1X PBS for 1 h at RT in a humidified chamber. After another three washes, the cells were incubated with FITC-conjugated secondary antibodies against anti-*P. salmonis* (#D-FP-SRS-IF, Ango) and anti-phalloidin (#A22287, Thermo Fisher) conjugated with Alexa Fluor^®^ 647. Both antibodies were diluted 1:200 in 3% BSA-1X PBS for 1 h at RT in a humidified chamber. Three more washes were performed, and incubation with DAPI solution (#62248, Thermo Fisher) was performed at a dilution of 1:1000 for 10 min at RT in a humidified chamber. Finally, the cells were washed once more with 1X PBS, and the coverslips were mounted with Vectashield Antifade Mounting Media (#H-1000, Vector lab., Burlingame, CA, USA) for confocal microscopy analysis.

### 2.5. Confocal Microscopy Analysis

The acquisition settings were the same in all treatments for the three independent plates. Images were taken using a TCS SP8 confocal fluorescence microscope (Leica, Wetzlar, Germany) with numerical aperture 1.4 × 100 oil objective, ×2 digital zoom (NA = 1.4; HC PL APO CS2 100×) and a z-step of 1 μm optical sections (velocity scan 600 Hz; resolution 1024 × 1024 pixels, equivalent to 58.13 μm × 58.13 μm). The following three laser wavelengths were used for DAPI (Ex 405 nm and Em 410–483 nm), FITC (Ex 488 nm and Em 502–621 nm), and Alexa Fluor^®^ 647 (Ex 638 nm and Em 650–776 nm), and signals were detected with an ultrahigh dynamic photomultiplier (PMT) spectral detector. Maximum intensity projections of confocal z-stack images of *P. salmonis* in whole nuclei (containing 17–22 stacks with resolution equivalent to 4.81–6.21 μm) were analyzed. All pictures and three-dimensional (3D) reconstructions of myotubes were performed using Leica Application Suite X v.3.5.5 software (Leica).

### 2.6. Library Construction and Sequencing

Myotubes were cultured in 12-well plates (8 × 10^5^ cells/well), and they were treated as previously mentioned. The RNA from myotubes was extracted using E.Z.N.A^®^ Total RNA kit (#R6834, Omega Bio-Tek, Norcross, GA, USA) following the manufacturer’s recommendations. The RNA collected was measured by fluorometry using the Qubit RNA BR assay kit (#Q10211, Thermo Fisher). Then, a capillary electrophoresis Fragment Analyzer™ Automated CE System (Advanced Analytical Technologies, Inc., Ames, IA, USA), was used to confirm the RNA integrity. RNA samples with RNA quality numbers (RQN) ≥ 8.5 were selected. A total of 12 cDNA libraries corresponding to 1 µg of RNA from three biological replicates (*n* = 3) of each condition (CTRL, CORT, INF, and C+INF) were generated using TruSeq™ RNA Sample Preparation kit v2, Set A (#RS-122-2001, Illumina, San Diego, CA, USA), according to the manufacturer’s protocol. Then, libraries were quantified using the KAPA Library Quantification Kit (#KK4824, Roche, Basilea, Switzerland), and their integrity was analyzed by a fragment analyzer. All libraries were sequenced using a paired-end strategy (2 × 100 bp) with the Illumina HiSeq 4000 platform by the Macrogen sequencing company (Seoul, South Korea). Raw read sequences were deposited in the Sequence Read Archive (SRA) (http://www.ncbi.nlm.nih.gov/sra; accessed date 31 May 2021) under the accession number PRJNA732666.

### 2.7. Raw Data Processing and RNA Sequencing Analysis

The transcriptional response of rainbow trout myotubes associated with CORT or C+INF was determined by RNA-seq after the previously mentioned treatments. Additionally, CTRL and INF, but without cortisol, were included as controls during the assay. All RNA-seq analyses were performed according to [25], with minor modifications. Briefly, raw reads were visualized using CLC Genomic Workbench 9.0 software. Then, the adapters, low-quality reads (Q < 30), and reads length <50 bp were discarded. High-quality reads were mapped onto the reference rainbow trout genome (Omyk_1.0, RefSeq assembly accession: GCF_002163495.1) which consists of 71,413-coding sequence (CDS) [26] using default mapping parameters: Mismatches = 2, minimum fraction length = 0.9, minimum fraction similarity = 0.8, and maximum hits per read = 5.

The unique mapped reads were used for differential expression analysis using empirical analysis of DGE incorporated in the CLC platform. Transcripts with absolute fold-change values > 2.0 and a false discovery rate (FDR)-corrected *p*-value < 0.05 were considered differentially expressed transcripts (DETs).

### 2.8. Functional Annotation and Gene Ontology Analysis

Based on the DETs, an ontological enrichment analysis was performed to obtain an overall view of the main biological processes, cellular components, molecular functions, and KEGG pathways that were affected during the trial.

Functional annotation was performed to obtain the gene ontology (GO) ID of each transcript, and a search against different fish databases, including rainbow trout, Atlantic salmon, Coho salmon, zebrafish (*Danio rerio*), and Atlantic cod (*Gadus morhua*), was performed with the BLASTx tool and the UniProt database (https://www.uniprot.org/; accessed date 6 December 2020). The enrichment analysis was performed by the DAVID-GO v.6.8 tool (https://david.ncifcrf.gov/; accessed date 23 January 2021) with the gene IDs of transcripts that were differentially expressed [27]. Standard settings for the DAVID analysis were gene count: 2 and EASE score: 1. The cut-off for the modified Fisher exact *p*-value was 3.30 × 10^−1^. Then, the resulting GO analysis was plotted in bubble charts.

### 2.9. Real-Time PCR Validation and Bacterial Gene Expression

The gene expression values in silico involved in enriched biological processes were validated by real-time PCR assays (RT-qPCR). Validation was performed with genes involved in the immune response (*nod1, adam15, ikba*), apoptosis (*hip1r, s30bl, rbl1*), and growth (*arhgap32, mprip, mylk3*).

RNA was extracted and quantified as previously mentioned, and 1 μg was reverse transcribed to cDNA using the QuantiTect^®^ reverse transcription kit (#205313, Qiagen, Hilden, Germany). The primers used to amplify the candidate genes were designed using Primer3 (https://primer3.ut.ee/; accessed date 3 February 2021), validated with NetPrimer (http://www.premierbiosoft.com/netprimer/; accessed date 3 February 2021), and synthesized by Integrated DNA Technologies, Inc. (San José, CA, USA).

An Mx3000P qPCR system (Stratagene, San Diego, CA, USA) was used for all RT-qPCR. The reactions contained 7.3 µL of 2X Brilliant II SYBR^®^ Green qPCR Master Mix (#600828, Agilent Technologies, Santa Clara, CA, USA), 0.2 µL of ROX reference dye (5 µM), 0.75 µL of each primer (250 nM) and 6 µL of cDNA (200 ng) in a 15 µL final volume. Furthermore, all RT-qPCR assays were performed using triplicates, no-reverse transcriptase control, and a no-template control. The list of primers used in this study is listed in Supplementary Table S1. Amplifications were performed with the following thermal cycling conditions: Initial activation at 95 °C for 10 min, followed by 40 cycles of 30 s at 95 °C, 30 s of Tm, and 30 s at 72 °C. A dissociation curve was generated to confirm the generation of a single PCR product. Gene expression analysis was performed using the geNorm program [28], combining the geometric average of two stable reference genes: 40S ribosomal protein 30S (*fau*) and β-actin (*actb*).

Additionally, the expression profiles of *P. salmonis* genes were analyzed to determine differences between infected and cortisol pretreated rainbow trout myotubes. RT-qPCR of pathogenesis-related genes of *P. salmonis*, including Dot/Icm (*dotA, dotB, icmE, icmK*), type 4 pili (*pilA, pilQ*), thiol-specific peroxidase (*ahpC*), and bacterial load (*23s*) was performed with total RNA from myotubes infected with *P. salmonis* (INF) and myotubes preincubated with cortisol and then infected with *P. salmonis* (C+INF). For this, the primer sequences used were obtained as follows: Dot/Icm [29], type 4 pili [30], *ahpC* [31], and *23s* [32]. The *succinate dehydrogenase A subunit* (*sdhA*) and *DNA gyrase B subunit* (*gyrB*) genes were used as normalizing genes, which were previously standardized by [33].

### 2.10. Statistical Analysis

The data are expressed as the mean ± standard error of the mean (SEM). Differences in means between groups were determined using one-way ANOVA followed by Tukey’s honestly significant difference posttest (HSD). Correlations between RNA-seq and RT-qPCR data were assessed through multiple linear regressions using Pearson correlation (r) and *p*-values. Data were accepted as significant at a *p*-value < 0.05. All statistical analyses were performed using GraphPad Prism v.9.0.

## 3. Results

### 3.1. Intracellular Location of P. salmonis between Rainbow Trout Myotubes with Cortisol and/or P. salmonis

**Figure 1:**
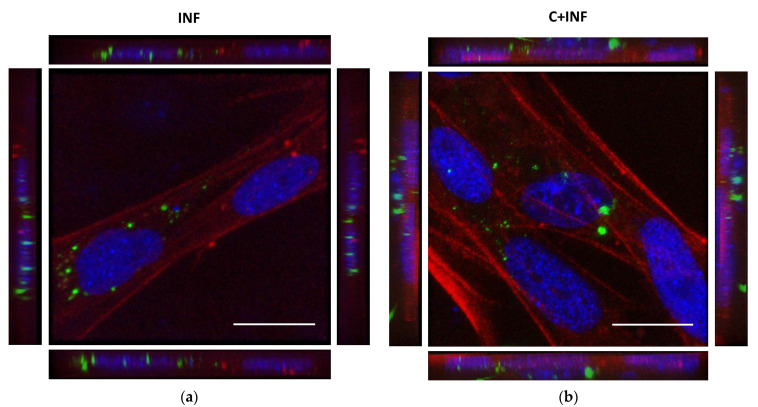
Intracellular location of *P. salmonis* between rainbow trout myotubes infection and cortisol pretreatment. Representative confocal images of the immunofluorescence staining of *P. salmonis* (green) and phalloidin (actin cytoskeleton, red). (**a**) Primary culture of rainbow trout skeletal muscle cells infected with *P. salmonis* strain LF-89 (MOI 50) for 8 h (INF). (**b**) Rainbow trout myotubes pretreated with 3 h of cortisol (100 ng/mL) followed by infection with *P. salmonis* (C+INF). Nuclei were stained with DAPI (blue). The images represent a maximum projection for the total nuclear volume. Scale bar: 20 µm.

The immunostaining for *P. salmonis* in rainbow trout myotubes was performed to detect its subcellular location. The images and 3D reconstructions of both INF (Figure 1a) and C+INF (Figure 1b) indicated an intracellular location of bacteria (in green) in muscle cells (in red) near the nuclei (in blue). No *P. salmonis* was detected in the CTRL and CORT groups (Supplementary Figure S1). The isolated images for all conditions are available in Supplementary Figure S1.

### 3.2. Transcriptomic Responses of Rainbow Trout Muscle Cells with Cortisol and/or P. salmonis

A total of 776,965,304 million readings were obtained from 12 cDNA libraries (Supplementary Table S2). After adapters were removed and low-quality readings were discarded, 704,979,454 high-quality readings were obtained (Supplementary Table S2). A total of 468,598,883 high-quality readings (70.25%) were specifically mapped against the 71,413 CDSs of the rainbow trout genome (Supplementary Table S3).

Differential expression analysis in silico was performed by specifically mapping the readings against a reference genome. A table was generated, indicating a total of 175 differentially expressed transcripts (DETs) for all comparisons and 124 upregulated and 51 downregulated genes (Table 1). In addition, the CTRL vs. INF presented 1 was unique, and 12 were shared DETs. For CTRL vs. CORT, 6 DETs were unique, and 40 were shared. For CTRL vs. C+INF, 11 were unique, and 27 were shared DETs. For CORT vs. INF, 6 were unique, and 33 were shared DETs. For INF vs. C+INF, 6 was unique, and 24 were shared DETs. Finally, CORT vs. C+INF no presented unique DETs, and 9 were shared.

### 3.3. Gene Ontology Enrichment Analysis of Treated Rainbow Trout Myotubes

**Figure 2:**
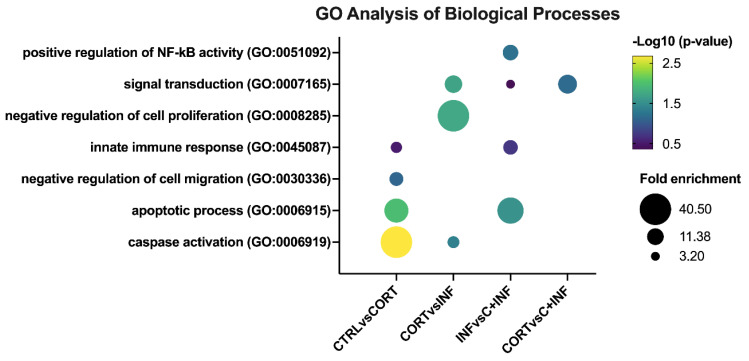
GO analysis of biological processes in rainbow trout myotubes treated with cortisol and/or infected with *P. salmonis*. The most representative and significant biological processes are represented in bubble plots and are sorted by group. The dot size indicates the fold enrichment associated with the process, and the dot color indicates the significance of the enrichment (−log10(the modified Fisher exact *p*-value)). Abbreviations: CTRL, control; CORT, cortisol; INF, infection; C+INF, cortisol+infection.

In the biological process component of GO analysis (Figure 2), the CTRL vs. CORT group presented the following GO terms: Activation of cysteine-type endopeptidase activity involved in the apoptotic process (or caspase activation), negative regulation of cell migration, apoptotic process, and innate immune response.

When comparing the CORT vs. INF groups, signal transduction, caspase activation, and negative regulation of cell proliferation were detected. In the comparison of the INF vs. C+INF group, the main biological processes were positive regulation of NF-kappaB transcription factor activity, signal transduction, apoptotic process, and innate immune response. However, no enrichment of biological processes for the CTRL vs. INF and CTRL vs. C+INF comparisons was observed. In the CORT vs. C+INF groups, only signal transduction was detected.

Regarding the cellular component of the GO analysis (Supplementary Figure S2), intracellular membrane-bound organelle, autophagosome, and cytosol were the most enriched terms in the CTRL vs. CORT and CORT vs. INF groups. Then, in the INF vs. C+INF groups, cytosol, endoplasmic reticulum membrane, and plasma membrane were detected. In the CTRL vs. INF groups, adherens junctions and cytosol were observed. In the CTRL vs. C+INF groups, the cellular components identified were early endosome membrane, endoplasmic reticulum membrane plasma membrane, and cytosol. Cytosol was enriched when comparing the CORT vs. C+INF groups.

The molecular function GO analysis revealed that phosphatidylinositol-3,4-bisphosphate binding, phosphatidylinositol-3,4,5-triphosphate binding, protein binding, ATP binding, metal ion binding, and protein binding were the most enriched terms in the CTRL vs. CORT groups (Supplementary Figure S3). When comparing the CORT vs. INF groups, the GO terms were ATP binding and protein binding. In the INF vs. C+INF group, protein binding and heat shock protein binding were detected. In the CTRL vs. INF groups, only protein binding was identified. Phosphatidylinositol-3-3,4,5-trisphosphate, phosphatidylinositol-3,4-biphosphate binding, and protein binding were detected in the CTRL vs. C+INF groups. Then, in the CORT vs. C+INF groups, only protein binding was detected. Finally, no enriched KEGG pathways were detected for any comparison.

### 3.4. Validation of RNA Sequencing Data by Real-Time PCR and Bacterial Gene Expression

**Figure 3:**
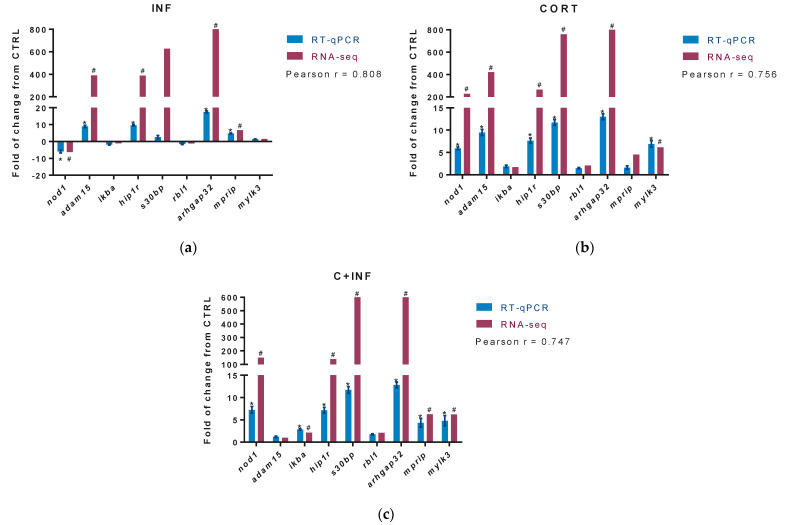
RT-qPCR validation of differentially expressed transcripts. The potential target genes selected for RT-qPCR validation of RNA-seq were *nod1, adam15, ikba, hip1r, s30bl, rbl1, arhgap32, mprip*, and *mylk3*. For RNA-seq, “#” indicates fold-change (absolute values > 2.0) and FDR corrected *p*-value (*p* < 0.05). (**a**) Validation between CTRL vs. INF. (**b**) Validation between CTRL vs. CORT. (**c**) Validation between CTRL vs. C+INF. For RT-qPCR, relative expression was normalized against *fau* and *actb*, and “*” indicates significant differences from the control (mean ± SEM, *n* = 3, *p* < 0.05). Pearson correlation (r) between RNA-seq and qPCR are also indicated (*p* < 0.05). Abbreviations: CTRL, control; CORT, cortisol; INF, infection; C+INF, cortisol+infection; *nod1*, Nucleotide-binding oligomerization domain-containing protein 1; *adam15*, Disintegrin and metalloproteinase domain-containing protein 15; *ikba*, NF-kappa B inhibitor alpha; *hip1r*, Huntingtin-interacting protein 1-related protein; *s30bp*, SAP30-binding protein; *rbl1*, Retinoblastoma-like protein 1; *arhgap32*, Rho GTPase activating protein 32; *mprip*, myosin phosphatase Rho interacting protein; *mylk3*, myosin light chain kinase 3; *fau*, 40S ribosomal protein S30; *actb*, β-actin.

The DETs from enriched biological processes of immune response (innate immune response (GO:0045087) and positive regulation of NF-kappaB transcription factor (GO:0051092)), apoptosis (caspase activation (GO:0006919) and apoptosis process (GO:0006915)), and growth (negative regulation of cell migration (GO:0030336) and negative regulation of cell proliferation (GO:0008285)) were identified in RNA-seq analysis. From these DETs, the top three in fold change were selected for RT-PCR. Figure 3a indicates the results of the validation in the experimental group infected with *P. salmonis*. Figure 3b displays the data for the group treated with cortisol. Figure 3c shows the validation obtained after pretreatment with cortisol and then infection with *P. salmonis*.

Our results present high correlations in the group infected with *P. salmonis* (r = 0.808), in the group treated with cortisol (r = 0.756), and in the group pretreated with cortisol and infected with *P. salmonis* (r = 0.747), between the expression values of those candidate genes used in the RNA-seq and RT-qPCR.

**Figure 4:**
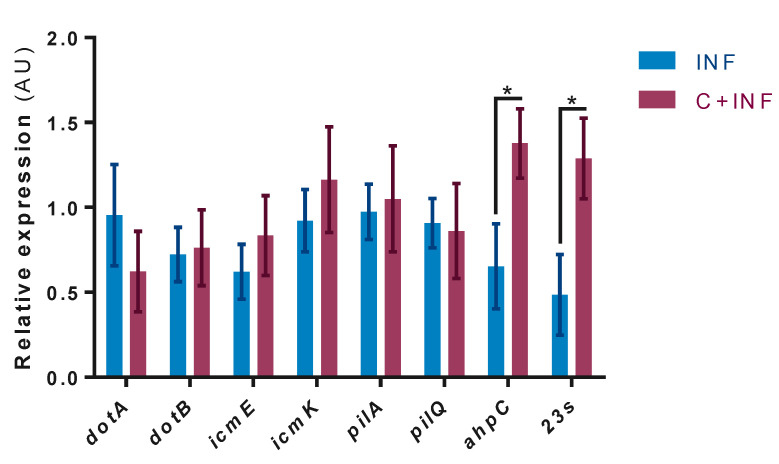
Bacterial gene expression between infected myotubes with cortisol pretreatment. Pathogenesis-related gene expression by RT-qPCR of *P. salmonis*: Dot/Icm (*dotA, dotB, icmE, icmK*), type 4 pili (*pilA, pilQ*), thiol-specific peroxidase (*ahpC*), and bacterial load (*23s*). Data were normalized against *sdhA* and *gyrB* and are expressed in arbitrary units (AU) as the mean ± SEM (*n* = 3). The “*” indicates significant differences between INF vs. C+INF (*p* < 0.05). Abbreviations: INF, infection; C+INF, cortisol+infection; *dotA*, deficient in organelle trafficking A; *dotB*, deficient in organelle trafficking B; *icmE*, intracellular multiplication E; *icmK*, intracellular multiplication K; a*hpC*, alkyl hydroperoxide reductase C; *sdhA*, succinate dehydrogenase A subunit; *gyrB*, DNA gyrase B subunit.

In the mRNA levels of *P.salmonis* genes, no expression was detected in the CTRL and CORT groups. The results indicate the significant upregulation of the *ahpC* and *23s* genes in the C+INF group compared to the INF group (Figure 4). No changes were observed in the other evaluated genes.

## 4. Discussion

RNA-seq is widely used to evaluate the transcriptomic immune response during several fish pathogen infections in vivo [34,35], but few studies have evaluated this transcriptomic response in vitro [36,37]. Here, we performed RNA-seq analysis to evaluate the effects of cortisol on rainbow trout myotubes infected with *P. salmonis*. The quality obtained from RNA-seq data was as expected, and the mapping results against the rainbow trout reference genome were in line with studies using this strategy [25,38]. According to these results, other recent reports in trout presented similar results using comparable parameters of RNA-seq analysis [39,40].

In myotubes treated with cortisol and/or infected with *P. salmonis*, GO enrichment analysis revealed innate immune response, apoptotic process, and negative regulation of cell proliferation as the most impacted biological processes. A recent study of RNA-seq on salmonid immune cells infected with *P. salmonis* observed similar processes [36], which could indicate that muscle cells respond similarly to infection. This is also consistent with the observations in skeletal muscle of salmon infected with these bacteria [41], and in in vitro and in vivo infections of rainbow trout skeletal muscle with *P. salmonis* LF-89 strains [18,20].

For validation, the selected genes involved in the immune response were *nod1, adam15*, and *ikba*. Nucleotide-binding oligomerization domain-containing protein 1 (nod1) is a cytosolic receptor that recognizes intracellular pathogen-associated molecular patterns via nuclear factor-kappa B (NF-kappaB), promotes proinflammatory cytokine transcription, and activates apoptosis by inflammatory caspases [42,43]. Disintegrin and metalloproteinase domain-containing protein 15 (adam15) is a transmembrane glycoprotein involved in immune cell recruitment to inflammation sites, among other processes [44,45]. Interestingly, no changes were detected in myotubes infected with cortisol pretreatment compared to infection or cortisol. This could suggest immunomodulation of cortisol during infection. During infection and proinflammatory responses, NF-kappaB inhibitor alpha (ikba) becomes phosphorylated, promoting ubiquitination and degradation, enabling NF-kB to translocate to the nucleus and activate the transcription of immune-related genes [46]. The immune response could be explained in part by immune cells detected in skeletal muscle [47], but notably, in this study, there was no infiltration of immune cells. Hence, our results suggest an intrinsic, innate immunity of myotubes during infection. Stress can modulate the immune response in fish [10], and we also detected differential regulation by cortisol. This is reflected by the apoptotic process enrichment observed during trials, for which the *hip1r, s30bp*, and *rbl1* genes were selected for validation. Huntingtin-interacting protein 1-related protein (hip1r) is a component of the endocytic machinery that interacts with the actin cytoskeleton and is involved in the intrinsic pathway of apoptosis [48]. The SAP30-binding protein (s30bp) is a member of the Sin3-associate polypeptide (SAP) complex, which regulates apoptosis by transcriptional corepressor of cell survival-related genes [49]. Retinoblastoma-like protein 1 (rbl1) is a key regulator of entry into cell division that acts as a transcription coactivator of glucocorticoid receptor-induced apoptosis [50]. These results are consistent with previous reports, revealing that cortisol-mediated stress can also regulate apoptosis and during infection. Most likely, *P. salmonis* could either interfere with proteins that inhibit the apoptosis process or activate intrinsic programmed cell death. Supporting these data, in rainbow trout myoblasts infected with *Flavobacterium psychrophilum*, apoptosis was induced through NF-κB signaling as a mechanism for nutrient acquisition and survival [51]. However, further analyses are required to comprehend the mechanisms by which this bacterium induces apoptosis.

Growth-related genes (*arhgap32, mprip, mylk3*) were evaluated because a negative regulation of cell proliferation was detected. Rho GTPase activating protein 32 (arhgap32) induces GTP hydrolysis of GTPases that control actin assembly of the cytoskeleton in cell growth [52]. Myosin phosphatase Rho interacting protein (mprip) binds to myosin phosphatase of actin fibers and regulates the cytoskeletal structure of muscle cells [53]. Myosin light chain kinase 3 (mylk3) encodes a protein kinase that phosphorylates myosin, promotes sarcomere formation, and increases the contractility of muscle [54]. It has been described that cortisol regulates muscle growth [55], but there is no information during infection in vitro. We found that perhaps either an imbalance of the proliferation/differentiation rates or alteration of the cytoskeleton were involved. During myogenesis in fish, a balance between proliferation and differentiation in muscle cells is necessary for optimal growth [56]. When one of these processes is affected, the other is compensated. In this case, a downregulation of proliferation occurred and was then compensated by an increase in differentiation. Previous studies have described that *P. salmonis* can affect the cytoskeleton during infection [57,58]. Supporting this, a decreased pattern of the actin cytoskeleton was presented following the immunostaining of infected rainbow trout myotubes compared to cortisol pretreated myotubes, indicating that *P. salmonis* could decrease the expression levels of genes related to the cytoskeleton structure of rainbow trout myotubes that were upregulated by cortisol.

Finally, we analyzed the expression of pathogenesis-related genes to determine whether cortisol has a synergistic effect. Surprisingly, two genes were upregulated: Large-subunit ribosomal (*23s*), a marker for bacterial load, and alkyl hydroperoxide reductase C (*ahpC*). The immunostaining of *P. salmonis* presented a similar pattern of bacterial abundance, which is consistent with the mRNA levels of *23s*. Otherwise, thiol-specific peroxidase catalyzes the reduction of hydrogen peroxide and organic hydroperoxides to water and alcohols, respectively [59]. This mechanism protects the bacterium against oxidative stress generated by the host’s immune cells, contributing to pathogenesis [60]. This is especially interesting because it supports the immune response of muscle cells. In contrast, a transcriptomic profile of *P. salmonis* infecting SHK-1 cells alternated between replicative and stationary phases that activated a stringent response [37]. Additionally, dual RNA-seq between *P. salmonis* and Atlantic salmon spleen/head kidney indicated metabolic amino acid dependency [61]. These data suggest that this bacterium could also uptake nutrients from skeletal muscle. Together, stress-induced cortisol production could negatively affect muscle cells and promote pathogenesis during infection.

## 5. Conclusions

In summary, for the first time, we described a transcriptomic response of myotubes treated with cortisol and/or infected with *P. salmonis* by inducing an innate immune response, apoptosis, and negative regulation of cell proliferation. Additionally, cortisol pretreatment differentially stimulated the bacterial gene expression of *ahpC* and *23s* in infected myotubes. Although few DETs were detected, these had significant changes in magnitude and were related to key processes for muscle growth and immunity. Our data suggest that fish muscle cells have an intrinsic immune response that is differentially regulated by cortisol. The information provided here will help us to understand the molecular mechanisms of fish muscle cells respond to infection, which could lead to pathogen outbreaks in skeletal muscle under stress conditions.

## Figures and Tables

**Table 1:** The number of differentially expressed transcripts (DETs) in rainbow trout myotubes treated with cortisol and/or infected with *P. salmonis*. The table showed the number of total DETs, upregulated, and downregulated between all comparisons. Abbreviations: CTRL, control; CORT, cortisol; INF, infection; C+INF, cortisol+infection.

Comparisons	Total DETs	Upregulated DETs	Downregulated DETs
CTRL vs. INF	13	9	4
CTRL vs. CORT	46	33	13
CTRL vs. C+INF	38	27	11
CORT vs. INF	39	25	14
INF vs. C+INF	30	24	6
CORT vs. C+INF	9	6	3
Total	175	124	51

## Data Availability

The commercial antibodies and reagents used in this study were listed in the Material and Methods section. The nucleotide sequences used in this study were collected from the National Center for Biotechnology Information (NCBI) GenBank repository. Raw read sequences obtained from sequencing were deposited in the Sequence Read Archive (SRA) (http://www.ncbi.nlm.nih.gov/sra; accessed date 31 May 2021) under the accession number PRJNA732666. The datasets generated and/or analyzed during the current study are not publicly available due to privacy or ethical restrictions but are available from the corresponding author on reasonable request.

## Supplementary Materials

The following are available online at https://www.mdpi.com/article/10.3390/ani11082399/s1, Supplementary Figure S1: Immunofluorescence of rainbow trout myotubes pretreated with cortisol and/or infected with *P. salmonis*, Supplementary Figure S2: GO analysis of cellular components in rainbow trout myotubes treated with cortisol and/or infected with *P. salmonis*, Supplementary Figure S3: GO analysis of molecular functions in rainbow trout myotubes treated with cortisol and/or infected with *P. salmonis*, Supplementary Table S1: List of primers, amplicon size, and annealing temperature used in the RT-qPCR validation, Supplementary Table S2. Sequencing data obtained by RNA-Seq with an Illumina HiSeq 4000. Supplementary Table S3: Supplementary Table S3. Mapping data.
