# Supplementary material for: RNA-Seq-Based Analysis of Cortisol-Induced Differential Gene Expression Associated with Piscirickettsia salmonis Infection in Rainbow Trout (Oncorhynchus mykiss) Myotubes
Source: Animals (Basel). 2021 Aug 13;11(8):2399. doi: 10.3390/ani11082399 (PMC8388646; doi:10.3390/ani11082399)
Supplement: Supplementary file 1 [file animals-11-02399-s001.zip › animals-1292681-supplementary/Supporting Information figures.pdf]

Supporting information figures

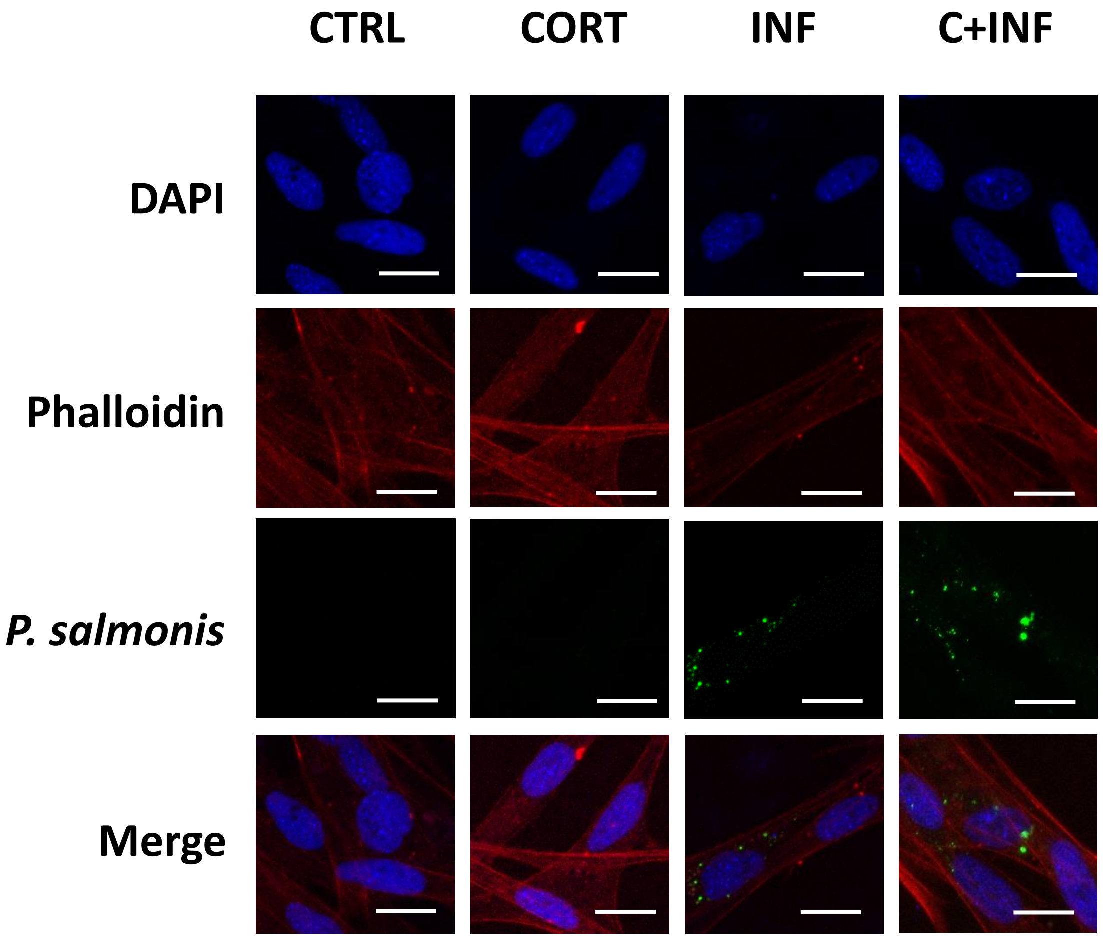

Supplementary Figure 1. Immunofluorescence of rainbow trout myotubes pretreated with cortisol and/or infected with *P. salmonis*. Representative confocal images of the immunofluorescence staining for DAPI (nuclei, blue), phalloidin (actin cytoskeleton, red) and *P. salmonis* (bacteria, green) in primary culture of rainbow trout skeletal muscle cells: control (CTRL), treated with 3 h of cortisol (100 ng/mL, CORT), infected with *P. salmonis* strain LF-89 (MOI 50) for 8 h (INF), and (B)

pretreated with cortisol and then infected with *P. salmonis* (C+INF). All images represent a maximum projection for the total nuclear volume. Scale bar: 20 µm.

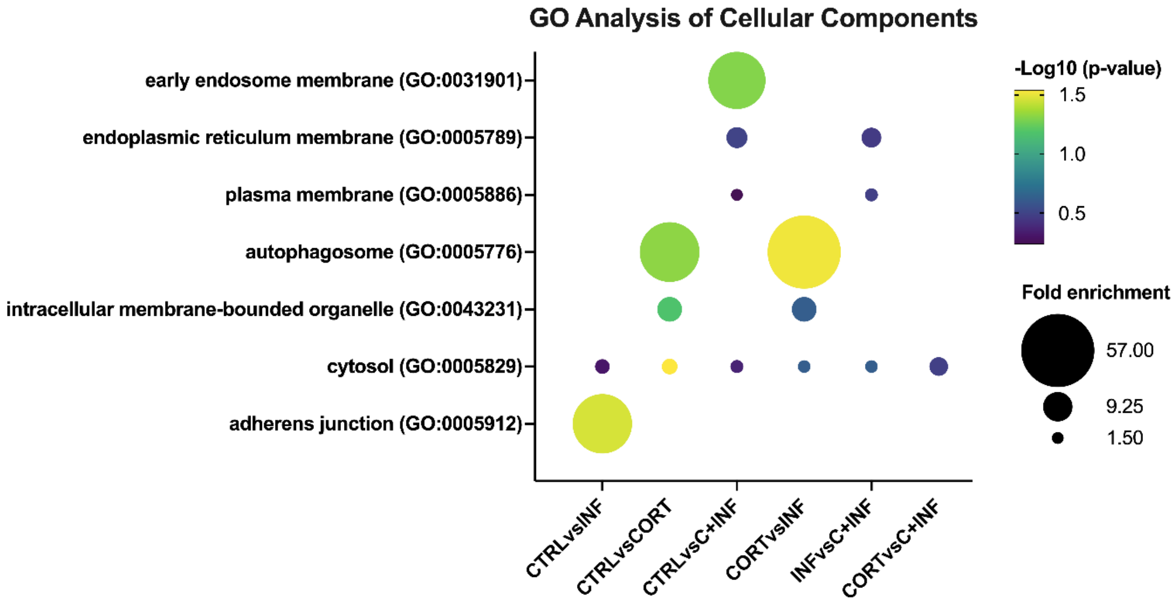

Supplementary Figure 2. GO analysis of cellular components in rainbow trout myotubes treated with cortisol and/or infected with *P. salmonis*. The most representative and significant cellular components are represented in the bubble plot and are sorted by each group. The dot size indicates the fold enrichment associated with the component, and the dot color indicates the significance of the enrichment (-log10(the modified Fisher exact p-value)). Abbreviations: CTRL: control; CORT: cortisol; INF: infection; C+INF: cortisol+infection.

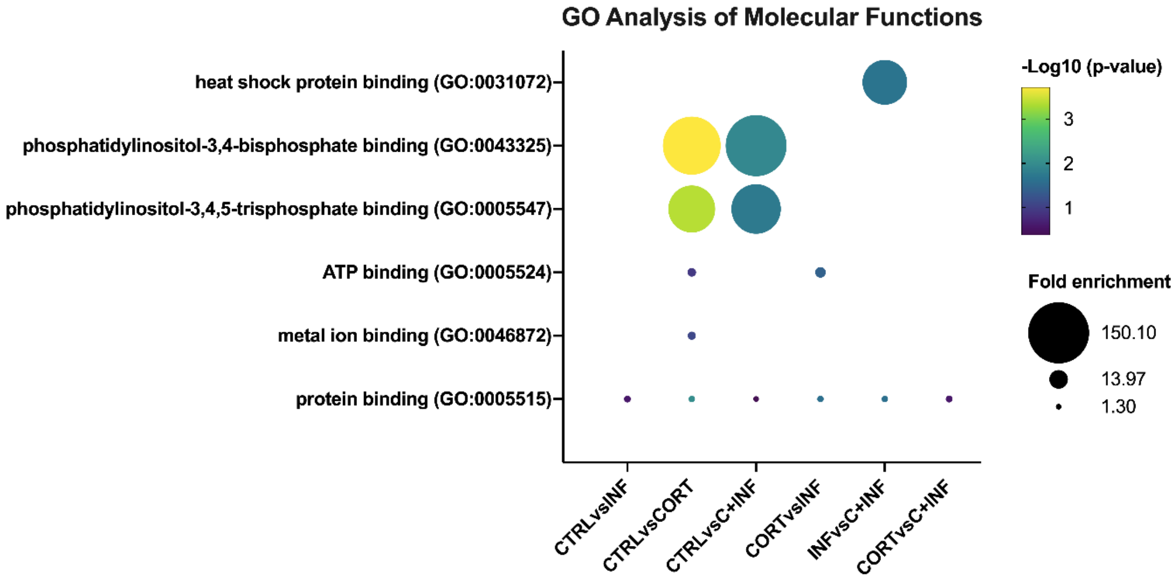

Supplementary Figure 3. GO analysis of molecular functions in rainbow trout myotubes treated with cortisol and/or infected with *P. salmonis*. The most representative and significant molecular functions are represented in bubble plots and are sorted by each group. The dot size indicates the fold enrichment associated with the function, and the dot color indicates the significance of the enrichment (-log10(the modified Fisher exact p-value)). Abbreviations: CTRL: control; CORT: cortisol; INF: infection; C+INF: cortisol+infection.
