# Supplementary material for: RNA-Seq-Based Analysis of Cortisol-Induced Differential Gene Expression Associated with Piscirickettsia salmonis Infection in Rainbow Trout (Oncorhynchus mykiss) Myotubes
Source: Animals (Basel). 2021 Aug 13;11(8):2399. doi: 10.3390/ani11082399 (PMC8388646; doi:10.3390/ani11082399)
Supplement: Supplementary file 1 [file animals-11-02399-s001.zip › animals-1292681-supplementary/Supporting Information tables modified.pdf]

Supporting information tables

Supplementary Table S1. List of primers, amplicon size, and annealing temperature used in the RT-qPCR validation.

Gene name	Sequence 5′ – 3′	Amplicon size (bp)	Tm (ºC)
nod1 **forward**	CTCCTCTTCGTTTGCCAGT	224	59
nod1 **reverse**	GAGCCCAGAATCAGCCTT		
adam15 **forward**	ATCCACATCCCACCACAAGT	211	61
adam15 **reverse**	CCAGACCAGTTAGAGCCACA		
ikba **forward**	TGCTCCTGGTCAGTAAA	177	55
ikba **reverse**	TGCTCCTGGTCAGTAAA		
hip1r **forward**	ATGGAGGAAATGGAGTCGC	197	60
hip1r **reverse**	ATGCTAGGCTTGGAGGGTT		
s30bp **forward**	AGAGGTAGAAGAAACAGAGGGC	235	61
s30bp **reverse**	TGAGAACAGAGCGACCAACT		
rbl1 **forward**	ATCTGGACCTGCGTTGAA	176	58
rbl1 reverse	ACACAGTTGCTTGCTTGAG		
mprip foward	TCAGTCCACACAGCATCTT	227	58
mprip reverse	TCATCTTTACCCCACCTACT		
arhgap32 foward	GTCATCAAACGCTACATCG	113	58
arhgap32 reverse	ACACACTCACTGGGAAAGAA		
mylk3 foward	AATGAGGGAGAGAGAGGAAG	142	57
mylk3 reverse	TCTGTCAGCAGGTAGTTC		
fau **forward**	CATTTAGGAGTTGGCGTTGG	134	57
fau **reverse**	CCAAGGTTGAAAAGCAGGAG		
actb **forward**	GCCGGCCGCGACCTCACAGACTAC	73	67
actb **reverse**	CGGCCGTGGTGGTGAAGCTGTAGC		

Supplementary Table S2. Sequencing data obtained by RNA-Seq with an Illumina HiSeq 4000. Twelve paired-end cDNA libraries were synthesized corresponding to the control group (CTRL), 3 h of treatment with 100 ng/ml of cortisol (CORT), infection with *P. salmonis* strain LF-89 (MOI 50) for 8 h (INF), and cortisol pretreatment followed by infection with *P. salmonis* (C+INF). Three biological replicates were performed per group (n = 3). In brackets, the percentage value of reads after trimming.

Library	N1 (raw data)	N1 (after trim)	N2 (raw data)	N2 (after trim)	N3 (raw data)	N3 (after trim)
**Reads CTRL**<br>**Avg Length**	67,911,730<br>101.0 bp	62,224,990 (91.63%)<br>89.4 bp	62,462,778<br>101.0 bp	57,528,904 (92.10%)<br>89.5 bp	65,182,754<br>101.0 bp	56,149,613 (86.14%)<br>89.1 bp
**Reads INF**<br>**Avg Length**	72,659,506<br>101.0 bp	66,613,265 (91.68%)<br>89.4 bp	58,492,120<br>101.0 bp	53,935,303 (92.21%)<br>89.5 bp	59,964,622<br>101.0 bp	54,307,173 (90.57%)<br>89.5 bp
**Reads CORT**<br>**Avg Length**	68,571,508<br>101.0 bp	62,755,326 (91.52%)<br>89.4 bp	58,692,224<br>101.0 bp	53,618,765 (91.36%)<br>89.4 bp	52,763,284<br>101.0 bp	45,465,247 (86.17%)<br>89.1 bp
**Reads C+INF**<br>**Avg Length**	74,388,534<br>101.0 bp	68,124,433 (91.58%)<br>89.4 bp	62,568,254<br>101.0 bp	57,467,203 (91.85%)<br>89.5 bp	73,307,990<br>101.0 bp	66,789,235 (91.11%)<br>89.5 bp
**Total reads (12 cDNA libraries)**	**raw data**<br>**776,965,304**	**after trim**<br>**704,979,457 (90.73%)**				

Supplementary Table S3. Mapping data. High-quality paired reads were mapped against the rainbow trout reference genome. Three biological replicates were performed per group (n = 3). Abbreviations: CTRL: control; CORT: cortisol; INF: infection; C+INF: cortisol+infection. In brackets, the percentage value of reads mapped against the reference genome.

Library	N1	N2	N3
**Reads CTRL**	42,482,594 (72.49%)	37,693,521 (68.89%)	36,100,759 (68.67%)
**Reads INF**	45,870,300 (73.11%)	35,150,238 (68.52%)	35,769,208 (69.07%)
**Reads CORT**	43,626,532 (73.88%)	34,741,302 (68.11%)	28,485,033 (66.72%)
**Reads C+INF**	46,549,288 (72.56%)	37,109,894 (67.71%)	45,020,214 (70.75%)
**Total mapped reads**	**468,598,883 (70.25%)**		
